# Rabies-induced behavioural changes are key to rabies persistence in dog populations: Investigation using a network-based model

**DOI:** 10.1371/journal.pntd.0007739

**Published:** 2019-09-23

**Authors:** Victoria J. Brookes, Salome Dürr, Michael P. Ward

**Affiliations:** 1 Sydney School of Veterinary Science, The University of Sydney, Camden, Australia; 2 School of Animal and Veterinary Sciences, Faculty of Science, Charles Sturt University, Wagga Wagga, Australia; 3 Veterinary Public Health Institute, University of Bern, Liebefeld, Switzerland; Imperial College London, UNITED KINGDOM

## Abstract

Canine rabies was endemic pre-urbanisation, yet little is known about how it persists in small populations of dogs typically seen in rural and remote regions. By simulating rabies outbreaks in such populations (50–90 dogs) using a network-based model, our objective was to determine if rabies-induced behavioural changes influence disease persistence. Behavioural changes–increased bite frequency and increased number or duration of contacts (disease-induced roaming or paralysis, respectively)–were found to be essential for disease propagation. Spread occurred in approximately 50% of model simulations and in these, very low case rates (2.0–2.6 cases/month) over long durations (95% range 20–473 days) were observed. Consequently, disease detection is a challenge, risking human infection and spread to other communities via dog movements. Even with 70% pre-emptive vaccination, spread occurred in >30% of model simulations (in these, median case rate was 1.5/month with 95% range of 15–275 days duration). We conclude that the social disruption caused by rabies-induced behavioural change is the key to explaining how rabies persists in small populations of dogs. Results suggest that vaccination of substantially greater than the recommended 70% of dog populations is required to prevent rabies emergence in currently free rural areas.

## Author summary

We investigated rabies spread in populations of 50–90 dogs using a simulation model in which dogs’ contacts were based on the social networks of three populations of free-roaming domestic dogs in the Torres Strait, Australia. Rabies spread would not occur unless we included rabies-induced behavioural changes (increased bite frequency and either roaming or paralysis that increased the number or duration of contacts, respectively). The model predicted very low case rates over long durations which would make detection challenging in regions in which there is already a high population turnover, increasing the risk of human infection and spread to other communities via dog movements. Spread also occurred in >30% of model simulations at low incidence for up to 200 days when 70% of the population was pre-emptively vaccinated, suggesting that higher vaccination coverage will be required to prevent rabies emergence in currently free rural areas, especially those in which dogs readily travel between communities.

## Introduction

Canine rabies is an ancient disease that has persisted in dog populations for millennia–well before urbanisation [1]. Increased understanding of rabies spread in communities with relatively small populations of dogs–such as those in rural and remote areas–could give insights about rabies persistence in non-urban areas, as well as inform prevention and control strategies in such regions.

Rabies virus is neurotropic and clinical manifestations of canine rabies can be broadly classified as the dumb form (characterised by progressive paralysis) and the furious form (characterised by agitated and aggressive behaviour; [2–4]). Although the mechanisms of rabies-induced behavioural signs are poorly understood [5], pathogen-influenced changes in host behaviour can optimise pathogen survival or transmission [6]. We hypothesise that rabies-induced behavioural changes promote rabies transmission in dog populations by influencing social network structure to increase the probability of effective contact. If so, this would enable rabies to spread in rural and remote regions.

Since 2008, rabies has spread to previously free areas of southeast Asia. Islands in the eastern archipelago of Indonesia, as well as Malaysia are now infected [7–10]. Much of this regional spread of canine rabies has occurred in rural and remote areas. Oceania is one of the few regions in the world in which all countries are rabies free. Recent risk assessments demonstrate that Western Province, Papua New Guinea (PNG) and northern Australia, are at relatively high risk of a rabies incursion [11, 12]. Dogs in communities in these regions are owned and roam freely. Population estimates in such communities are often low; for example, median 41 dogs (range 10–127) in Torres Strait communities (*pers comm*: annual surveys conducted by Queensland Health, and Brookes et al. [13]) and median 100 dogs (range 30–1000) in Western Province Treaty Villages (*pers comm*: annual surveys conducted by the Australian Commonwealth Department of Agriculture). Canine rabies might have a low probability of maintenance in domestic dogs in these communities due to their small population sizes, but if continued transmission occurs–particularly over a long duration–then spread to other communities or regional centres and regional endemicity might occur.

GPS telemetry data from small populations of dogs (< 50 dogs) in the Torres Strait have recently been collected [13]. Such data has been used to describe contact heterogeneity in animal populations, and has been used in models to provide insights about disease spread and potential control strategies [14–16]. The effect of contact heterogeneity on disease spread is well-researched and models can provide useful insights about disease control strategies in heterogeneously mixing populations [17–19]. Most recently in the context of rabies, Laager et al. [20] developed a network-based model of rabies spread using GPS telemetry data from dogs in urban N’Djamena, Chad. Other models of rabies-spread in which parameters that describe contact heterogeneity were derived from telemetry data include canine [21] and raccoon models [22, 23]. Patterns of contacts are likely to be altered by the behavioural effects of clinical rabies. Although Hirsch et al. [22] demonstrated that seasonal patterns of rabies incidence in raccoons could be explained by changes in social structure due to normal seasonal behavioural change of the hosts, the influence of rabies-induced behavioural changes on social structure has neither been researched nor explicitly incorporated in simulation models in any species.

Here, our objective was to investigate the probability, size and duration of rabies outbreaks and the influence of rabies-induced behavioural changes on rabies persistence in small populations of free-roaming dogs, such as those found in rural communities in PNG and northern Australia. We also investigate the effect of pre-emptive vaccination on rabies spread in such populations.

## Methods

### Disease model and study overview

**Fig 1:**
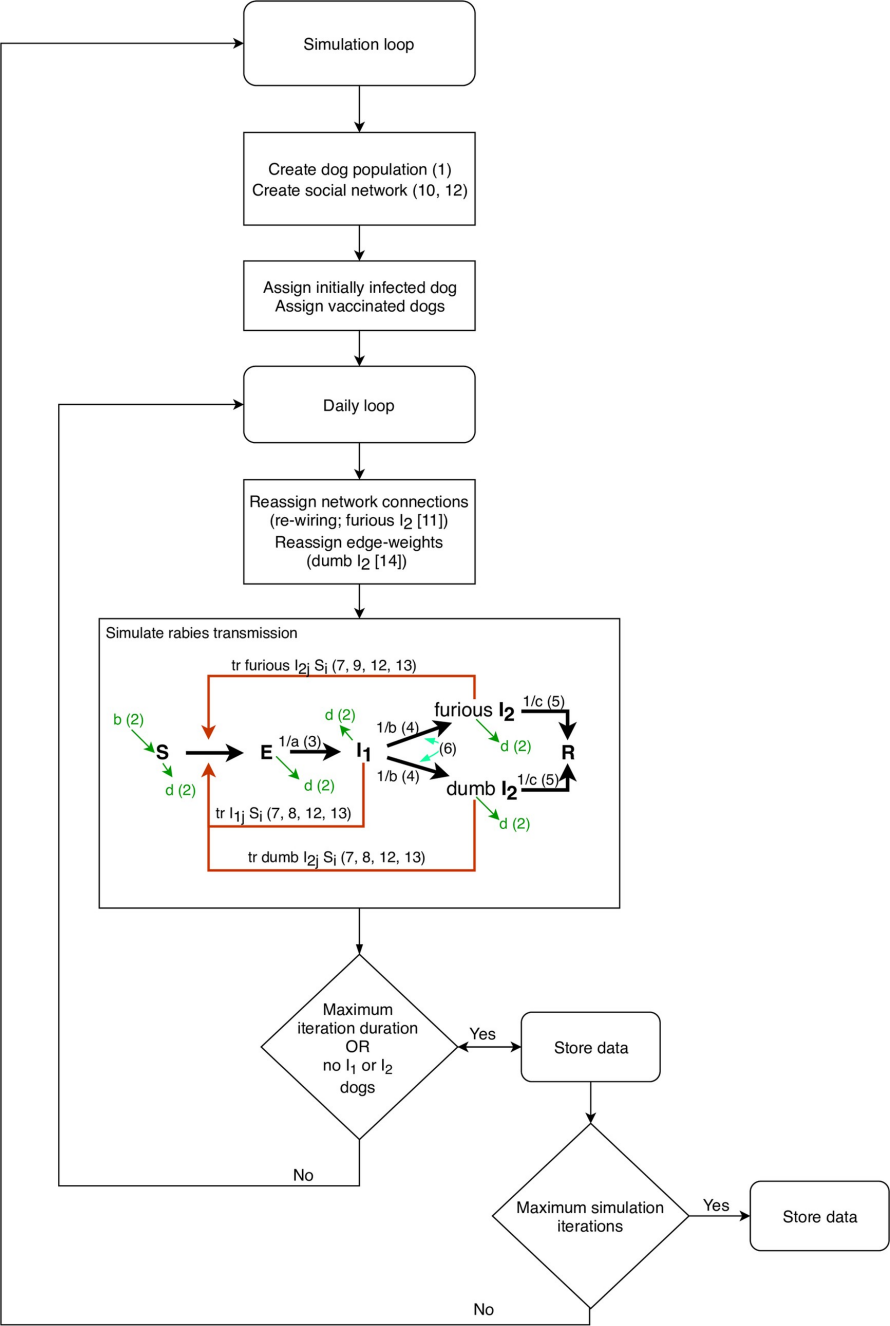
Process diagram of agent-based rabies spread simulation model in a population of dogs. S = susceptible state, E = latently infected state, I1 = pre-clinical infectious state, I2 = clinical state, R = dead state, tr = transmission, b = births, d = deaths, a = incubation period, b = pre-clinical infectious period, c = clinical period, j = individual infectious dog, i = individual susceptible dog. Numbers in parentheses are references to parameter values in Table 2.

We developed an agent-based, stochastic, mechanistic model to simulate social networks of free-roaming domestic dogs and the subsequent transmission of rabies between individual dogs within these networks following the latent infection of a single, randomly-assigned dog (Fig 1). The structure of the social networks was based on three empirically-derived networks of spatio-temporal associations between free-roaming domestic dogs in three Torres Strait Island communities (Table 1); Kubin, Warraber and Saibai [13]. The progression of rabies infection in a susceptible dog was simulated in daily time-steps and followed an SEI_1_I_2_R process (rabies infection status: susceptible [S], latent [E], pre-clinical infectious [I_1_], clinical [I_2_] and dead [R]).

**Table 1:** Properties of simulated networks of size 50–90 nodes (dogs) and their similarity to empirical networks of spatio-temporal association of dogs, tested by Mann-Whitney U (MWU) and Kolmogorov-Smirnoff (KS), in three island communities in the Torres Strait, Australia.

Property		Kubin	Saibai	Warraber
Simulated degree	median (range)	16 (7—22)	13 (5—16)	18 (7—20)
	MWU test P value (se)	0.28 (0.004)	0.28 (0.004)	0.29 (0.004)
	KS test P value (se)	0.82 (0.007)	0.84 (0.007)	0.82 (0.007)
Simulated edge-weight	median (95% range)	0.0004 (0—0.28)	4.1 x 10^−7^ (0—0.28)	0.0026 (0—0.42)
	MWU test P value (se)	0.30 (0.004)	0.30 (0.004)	0.32 (0.004)
	KS test P value (se)	0.83 (0.007)	0.87 (0.006)	0.86 (0.006)
Re-wiring probability, *ρ*		0.38	0.44	0.40
Average shortest path length (se)		1.84 (0.002)	1.80 (0.002)	1.95 (0.003)
Global clustering coefficient (se)		0.38 (0.002)	0.38 (0.001)	0.31 (0.001)
Small world index (se)		2.64 (0.01)	2.61 (0.01)	2.02 (0.01)

se = standard error

Rabies virus transmission from an individual infectious (*j*) to an individual susceptible (*i*) dog is described by Eq 1, in which the daily probability of contact between a pair of such dogs was calculated based on the edge-weight between the pair (*E*_*ij*_), which is the proportion of a 24 hour period during which that pair of dogs is spatio-temporally associated (in the event of no network connection, *E*_*ij*_ = 0). Transmission of rabies further depends on the probability of a bite (*P*_*j*_*)* by the infected dog conditional on its infection status (I_1_ or I_2_), and the probability of subsequent infection of the susceptible dog (*T*_*i*_). Generation of the social network and estimation of the parameters associated with the dog population dynamics and rabies epidemiology are described below, and parameter values are shown in Table 2. Maximum iteration duration was 3 years.

**Table 2:** Parameters used to describe canine rabies epidemiology in a simulation model of rabies spread in a dog population, to determine the influence of social structure on outbreak duration and number of rabies-infected dogs.

	Parameter	Distribution	Parameters	GSA value range	Source
1	Population size	uniform	range = 50—90	50—90	Empirical data [13]
2	Daily per capita birth/death rate	uniform	range = 0.8x10^-3^—1.2x10^-3^	0.8x10^-3^—1.2x10^-3^	Empirical data [29]
3	Incubation period (days)	Lognormal	μ = 2.76-3.13, δ = 0.54-0.75	7.4—65.8 days	[31]
4	Pre-clinical infectious period (days)	gamma	shape = 0.89-3.79, rate = 0.20-1.16	0.1—5.7 days	[30]
5	Clinical period (days)	gamma	shape = 2.49-3.56, rate = 0.81-1.19	1.1—10.2 days	[32]
6	Probability develops furious form	uniform	range = 0.2—0.5	0—0.5	[33–37, 40]
7	Probability of infection following bite	uniform	range = 0.4—0.52	0.4—0.52	[32]
8	Bite probability (pre-clinical and dumb)	uniform	range = 0.69 x 10^−2^—0.69 x 10^−1^	0.69 x 10^−2^—0.69 x 10^−1^	Empirical data [12]
9	*Bite probability (furious)	uniform	range = 0.69 x 10^−1^—0.69	0.69 x 10^−1^—0.69	Authors’ assumption, [32][32][32][32]
10	Initial ‘re-wiring’ probability, *ρ*	empirical	Community specific	0.38—0.44	See Table 1, re-wiring probability, *ρ*
11	*Probability of subsequent ‘re-wiring’ during the clinical phase	uniform	range = 0.05–0.25	0—0.25	Authors’ assumption
12	Degree distribution	empirical	Community specific	5—22	See Table 1, simulated degree [13]
13	Edge-weight distribution (spatio-temporal association)	empirical	Community specific	4.1 x 10^−7^—0.42	See Table 1, simulated edge-weight [13]
14	*Proportional increase in edge-weight during the clinical phase	uniform	range = 0—0.8	0—0.8	Authors’ assumption

sd = standard deviation, GSA = global sensitivity analysis.

*Rabies-induced behavioural changes.

$$\text{Probability}\;\text{of}\;\text{rabies}\;\text{transmission}=E_{íj}\;\cdot \;P_{j}|Status\;\cdot \;T_{i}$$ Eq 1

Model outputs included distributions of the predicted duration of outbreaks (defined as the number of days from the introduction of one latently infected dog to the day on which infected dogs were no longer present), the total number of rabies-infected dogs during the outbreak and the effective reproductive number, *R*_*e*_, during the first month following incursion (mean number of dogs infected by all dogs that were infected during the first month).

Initially, rabies was simulated in each of the three community networks and the predicted outputs from each model were compared between each other. Statistical tests were used to determine the number of iterations required to achieve convergence of output summary statistics (described below). Global sensitivity analysis using the Sobol’ method (described below) was used to investigate the relative influence of all input parameters on model outputs. To observe the influence of rabies-induced behavioural changes, model outputs from simulations of rabies spread in each of the three community networks with and without parameters associated with rabies-induced behavioural changes were compared. Finally, the impact of pre-emptive vaccination was investigated by randomly assigning rabies immunity to a proportion of the population (10–90%; tested in 10% increments) prior to incursion of the rabies-infected dog in each iteration.

### Social network generation

Prior to each iteration, a modified Watts Strogatz algorithm generated a connected, undirected small-world network of 50–90 dogs with network characteristics that reflected the empirical networks of the dog populations in Saibai, Warraber and Kubin communities, as follows [13, 24–26]. Consistent with the terminology used in our previous description of these networks [13], dogs are *nodes*, connections between dogs are *edges*, the proportion of spatio-temporal association (within 5m for at least 30s) between a pair of connected dogs in each 24 hour period is represented as *edge-weight*, and *degree* refers to the number of network connections for an individual dog. *Re-wiring* refers to re-assignment of an individual dogs connections in the network.

A regular ring lattice was constructed with *N* nodes in which *N* was randomly selected from a uniform distribution of 50–90. Each node was assigned *K* degrees, which was randomly selected from the respective empirical degree distribution of the community represented by the simulation. Each node (*n*_*i*_) was connected to *K*_*i*_/2 (rounded to the nearest integer) nearest neighbours in the ring lattice in a forward direction, then all nearest neighbours in a backward direction until *K*_*i*_ was achieved. Existing edges were then re-wired (the edge was disconnected from the nearest neighbour and reconnected to a randomly selected node) following a Bernoulli process (probability ρ) to achieve an average shortest path-length expected in an equivalent-sized Erdõs-Réyni graph in which nodes are connected randomly, whilst maintaining the empirical degree distribution of the community represented by the simulation [27]. Edges were then weighted according to the mean expected duration of association between pairs of dogs as a proportion of daily time, and were randomly selected from the respective empirical edge-weight distribution of the community represented by the simulation. Parameters that describe the empirical networks and their derivation are presented in Brookes et al. [13].

Networks simulated with the modified Watts Strogatz algorithm were tested for similarity to the empirical networks prior to use of the algorithm in the model (Table 1). Degree and edge-weight distributions were compared to those of the empirical networks using the Mann-Whitney U and Kolmogorov-Smirnoff tests, to assess similarity of median and shape of simulated distributions, respectively. Mean small-world indices were calculated according to Eq 2 in which *C* is the global clustering coefficient, *L* is the average shortest path length, *s* denotes a simulated network and *r* denotes an Erdos-Reyni random network of equivalent mean degree [28]. A small-world index >1 indicates local clustering, consistent with the empirical network structures. Network similarity tests were conducted on 1000 simulated networks for each community.

$$\text{Small-world}\;\text{Index}=\frac{C_{s}}{C_{r}}/\frac{L_{s}}{L_{r}}$$ Eq 2

### Parameter estimation

Parameters that were used to describe the dog populations and rabies epidemiology in the model are listed in Table 2.

#### Population dynamics

Population size was selected from a uniform distribution based on the expected size of dog populations in Torres Strait communities (*pers comm*: annual surveys conducted on behalf of Queensland Health, [13]). Births and deaths were included in the model with a daily per capita rate to maintain population size. Given population dynamics of free-roaming domestic dogs in similar communities in the region [29] and field-data from the Torres Strait communities, overall annual population turnover was 30–45% in the model.

#### Duration of latent, pre-clinical infectious and clinical periods

A distribution to describe the duration of the pre-clinical infectious period (I_1_) was parameterised by fitting a gamma distribution to data from an experimental study reported by Fekadu et al. [30] in which rabies virus was detected in the saliva of 12 dogs up to 13 days prior to the onset of clinical signs. In the model, the median pre-clinical infectious period was 1.4 days (95% range 0.1–5.7 days).

We did not find peer-reviewed literature that specifically described the latent period (E). Therefore, a lognormal distribution to describe the incubation period was reproduced from a study in which the date of exposure and onset of clinical signs were extracted from 92 case-records of rabies-infected dogs between 1948 and 1954 in Japan [31]. We included uncertainty in the distribution parameters (mean and standard deviation); in the model the median incubation period was 22.2 days (95% range 7.4–65.8 days). The latent period was then simulated for each infected dog in the model using a randomly selected incubation period and pre-clinical infectious period.

We reproduced a gamma distribution to describe the duration of the clinical period, derived from a study in which the case-histories of suspected rabies-infected animals were reconstructed [32], and included uncertainty in distribution parameters (shape and rate). In the model, median clinical period was 3.29 days (95% range 1.1–10.2 days) and all rabies-infected dogs died.

#### Proportion of dogs that develop furious rabies

In a study in Thailand, 79% of dogs were assessed as having the furious form of rabies, and similarly in a study in Zimbabwe, Foggin [33] found that 75% of rabid dogs submitted for laboratory examination showed signs consistent with the furious form. However, the proportion of dogs in which the furious form occurs is likely over-estimated in observational studies–dogs exhibiting furious rabies are more likely to roam and bite and are consequently most likely to be recognised and reported as suspect cases. Experimental studies suggest that the proportion of dogs that develop the furious form is less than 50% [34–36]. Therefore, we described the probability of developing the furious form of rabies using a uniform distribution with a conservative range (0.2–0.5). It should also be noted that whilst most dogs predominantly show one form of rabies, some dogs show signs attributable to both forms [4, 33, 37]. For model simplification, we assigned only one form of rabies in a clinical period.

#### Probability of a bite and subsequent rabies infection

We made the assumption that the incidence of bites by dogs in the pre-clinical infectious period or clinical period with the dumb form of rabies would reflect the background dog-bite incidence in the communities. Therefore, we assigned a daily probability of bite by an individual dog in these stages of infection according to a uniform distribution derived from a survey of free-roaming dogs in similar communities in the region (0.69 x 10^−2^–0.69 x 10^−1^;[12]). To reflect increased aggression and account for uncertainty about this parameter, the daily probability of a bite by a dog with the furious form of rabies in the clinical period was increased by an order of magnitude (0.69 x 10^−1^–0.69). This higher range is also consistent with the distribution of the duration of the clinical period and the findings regarding the number of bites in previous studies [38, 39].

#### Network stability

Altered behaviour during the clinical period is likely to disrupt network contacts. For example, progressive paralysis leading to recumbency might increase the duration of association between potential contacts, and wandering might change network associations (which would increase the total number of contacts during the clinical period). Anecdotally, these behaviours are associated with the dumb and furious forms of rabies, respectively [4]. In a study of the behaviour of 24 community dogs with rabies in Zimbabwe [37], the odds of wandering were higher in dogs with the furious form compared to those with the dumb form (odds ratio 3.44), but overall findings were inconclusive, perhaps due to insufficient sample size (95% CI 0.60–19.64). In the model, network disruption was parameterised using a possibility to increase edge-weight to account for an increased duration of association between a dog and its existing contacts, and the wandering was parameterised using a possibility to ‘re-wire’ a dog’s network connections to other dogs during each 24 hour period of the clinical period of infection. For model simplification, we assigned these changes in network connections to dogs with the dumb and furious forms, respectively, and distributions for these parameters were wide to reflect uncertainty.

#### Parameters associated with rabies-induced behavioural changes

Three of the parameters described in the sections above–increased duration of contact (edge-weight), wandering (‘re-wiring’) and increased bite probability–were specifically associated with rabies-induced behavioural changes (Table 2). Each of these changes occurred only during the clinical period in the model and were either associated with the furious form (an increased bite probability and changed network associations due to altered mentation and aggression ('re-wiring') or the dumb form (a possible increase in the duration of association with existing network contacts due to progressive paralysis ('edge-weight').

### Sensitivity analysis

Variance-based GSA using the Saltelli method was used to determine which parameters most influenced the variance of outputs and was implemented in this study using the SALib module in Python [41]. The sequence of events were: parameter sampling to create a matrix of parameter sets for each iteration (parameter ranges are listed in Table 2), simulation using the parameter sets to obtain model output (duration of outbreaks, the total number of rabies-infected dogs and the mean monthly effective reproductive number, *R*_*e*_), and estimation of sensitivity indices (SIs) to apportion output variance to each parameter. Mean monthly *R*_*e*_ was used as the output of interest in relation to *R* for Sobol’ analysis, to remove the strong influence of incubation period on *R*_*e*_ in the first month.

To separate the influence of stochasticity from the variation associated with each parameter, the random seed was also included in the Sobol’ analysis [42]. The seed value for each iteration was selected from the parameter set (uniform distribution, 1–100).

First-order and total-effect SIs were estimated for each parameter, representing predicted output variance attributable to each parameter without and with considering interactions with other inputs, respectively. SIs were normalised by total output variance and plotted as centipede plots with intervals representing SI variance. Model output variance is most sensitive to inputs with the highest indices.

### Convergence testing

The number of iterations required to achieve sufficient convergence of summary measures was estimated using the following method.

Key output measures–the number of rabid dogs and the duration of outbreaks (days)–were recorded from 9,999 iterations of the model divided equally between all three communities. Ten sets of simulated outputs of an increasing number of iterations (1–5000) were sampled; for example, ten sets of outputs from 1 iteration, 10 sets of outputs from 2 iterations, 10 sets of outputs from 3 iterations, and so on. The mean number of rabies-infected dogs and outbreak duration was calculated for samples in each set. The coefficient of variation (CV; standard deviation/mean) of these sample means was then calculated for each set. With increasing iterations, the variation in sample mean between sets decreases and the CV approaches zero. The number of iterations was considered sufficient to indicate model output stability when 95% of the previous 100 iteration sizes CV was < 0.025.

## Results

In this section, we initially describe and compare the predicted outputs from simulations of rabies incursions in each empirical network (‘Community simulations’). This is followed by a comprehensive investigation of the influence of all model parameters on key outputs of interest: outbreak duration, number of infected dogs and the monthly effective reproductive ratio *Re* (‘Global sensitivity analysis’). We then describe the predicted effects of behavioural changes in more detail (‘Rabies-induced behavioural changes’), and finally, investigate the effect of pre-emptive vaccination (‘The impact of pre-emptive vaccination’).

### Community simulations

Each community simulation comprised 10,000 iterations (more than sufficient to achieve convergence of summary output statistics without limiting computational time [S1 Fig]).

Predicted outputs are shown in Table 3. The proportion of iterations in which a second dog became infected was greater than 50% in Kubin and Warraber communities, and 43% in Saibai. In these iterations, predicted median and upper 95% duration of outbreaks were longest in Warraber and shortest in Saibai (median: 140 and 78 days; 95% upper range 473 and 360 days, respectively). In the Warraber simulations, 0.001% of iterations reached the model duration limit of 1095 days. The number of infected dogs was reflected in the *R*_*e*_ estimates in the first month: 1.73 (95% range 0–6.0), 2.50 (95% range 1.0–7.0) and 3.23 (95% range 1.0–8.0) in Saibai, Kubin and Warraber communities, respectively. The rate of cases during these outbreaks was 2.4 cases/month (95% range 0.6–7.6), 2.0 cases/month (95% range 0.4–6.5) and 2.6 cases/month (95% range 0.5–8.0) in Saibai, Kubin and Warraber communities, respectively.

**Table 3:** Predicted outputs of rabies spread in incursions in which > 1 dog was infected in simulated networks of dog populations (size 50–90) based on the empirical networks of spatio-temporal association of dogs in three island communities in the Torres Strait, Australia.

Community	Rabies-infected dogs, median (95% range, maximum)	Duration (days), median (95% range; maximum)	Proportion > 1 dog infected
Kubin	6 (2–49; 80)	97 (16–360; 874)	0.51
Saibai	4 (2–30; 58)	78 (13–327; 1030)	0.43
Warraber	9 (2–60; 89)	140 (20–473; 1095)	0.58

### Global sensitivity analysis

**Fig 2:**
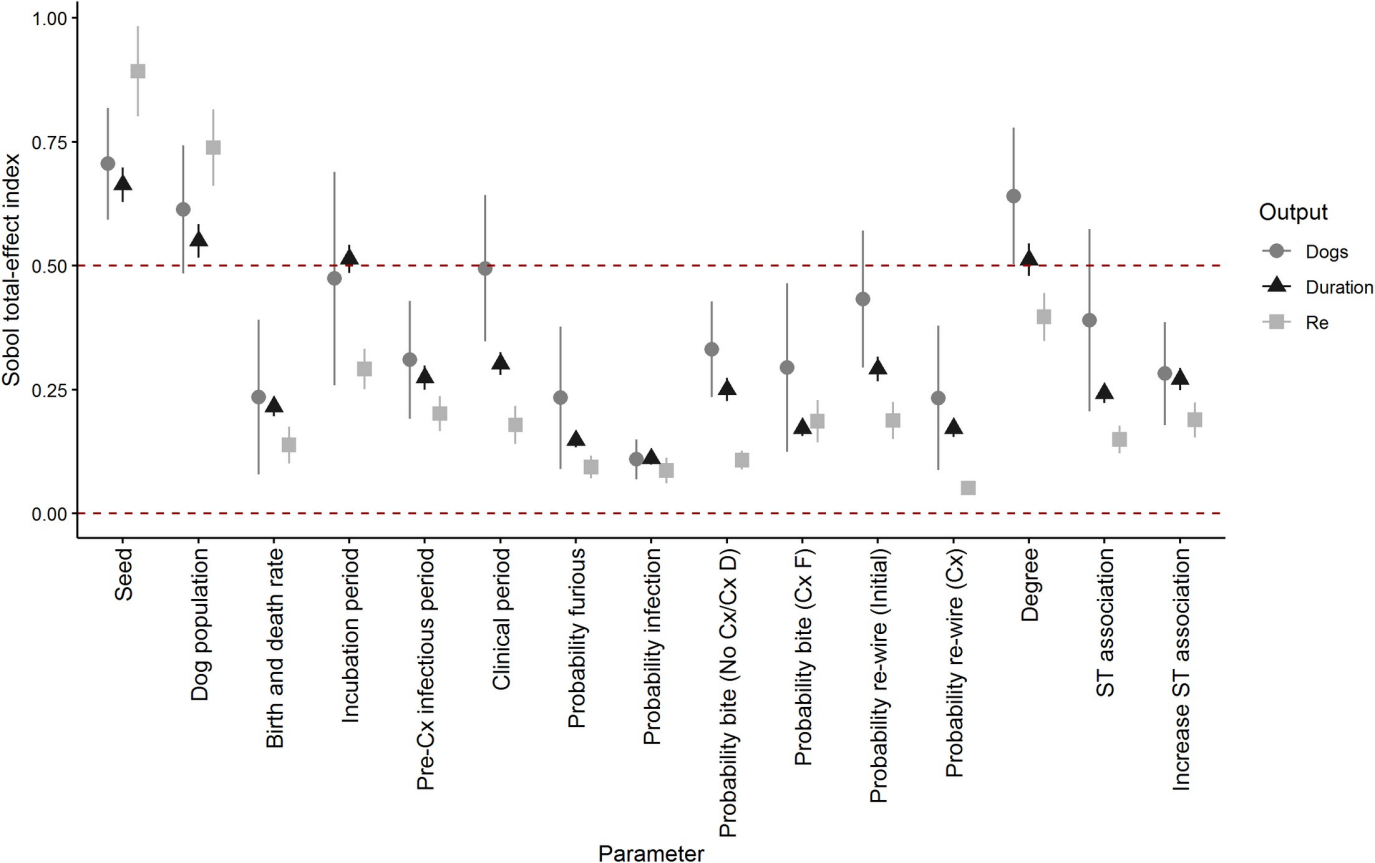
Total effect Sobol’ indices of parameters’ influence on the duration, number of rabies-infected dogs and mean monthly effective reproductive ratio of predicted rabies outbreaks following incursions of rabies in a simulated networks of dog populations based on the empirical networks of spatio-temporal association of dogs in three island communities in the Torres Strait, Australia. Cx = clinical signs, ST = spatio-temporal. Bars indicate 95% confidence intervals.

Fig 2 shows plots of the Sobol’ total-effect sensitivity indices (SI) of parameters for outbreak duration, number of infected dogs and the monthly effective reproductive ratio *R*_*e*_. S2 Fig shows Sobol’ first-order effect SIs, which are low relative to the total-effect SIs for all outcomes. This indicates that interactions between parameters are highly influential on output variance in this model and therefore, we focus on the influence of parameters through their total effects. As expected, the total-effect SI of the seed was highest–it was associated with > 50% of the variance for all outcomes–because it determines the random value selected in the Bernoulli processes that provide stochasticity to all parameters. The influence of the seed is not presented further in these results.

Incubation period, the size of the dog population and the degree of connectivity were highly influential on outbreak duration (total-effect SI 0.51, 0.55 and 0.51, respectively). All parameters were influential on the predicted number of rabid dogs (total effect SIs > 0.1). The size of the dog population, incubation and clinical periods, and degree had greatest influence (total effect SIs > 0.5). Dog population size and degree of association were most influential on predicted mean monthly *R*_*e*_ (total effect SI 0.74 and 0.40, respectively).

Of the community-specific parameters (population size, degree and edge-weight distributions, birth and death rates, and initial probability of re-wiring), dog population size and the degree consistently had the greatest influence on each predicted output’s variance. Of network parameters other than degree, the probability of wandering (‘re-wiring’) during the clinical phase (furious form) was markedly less influential on predicted mean monthly *R*_*e*_ than initial ‘re-wiring’ (total effect SIs 0.051 and 0.19, respectively) or either parameter associated with spatio-temporal association (edge-weight; both total effect SIs > 0.15). The influence of the increased probability of a bite by a dog in the clinical period (furious form) on predicted mean monthly *R*_*e*_ was greater compared to the pre-clinical or clinical (dumb-form) bite probability (total-effect SI 0.19 relative to 0.11). The size of the relative influence of these parameters on outbreak duration or number of rabies-infected dogs was reversed and less marked. Birth and death rate consistently had a moderate influence on all outputs (total-effect SI 0.20–0.24).

### Rabies-induced behavioural changes

**Fig 3:**
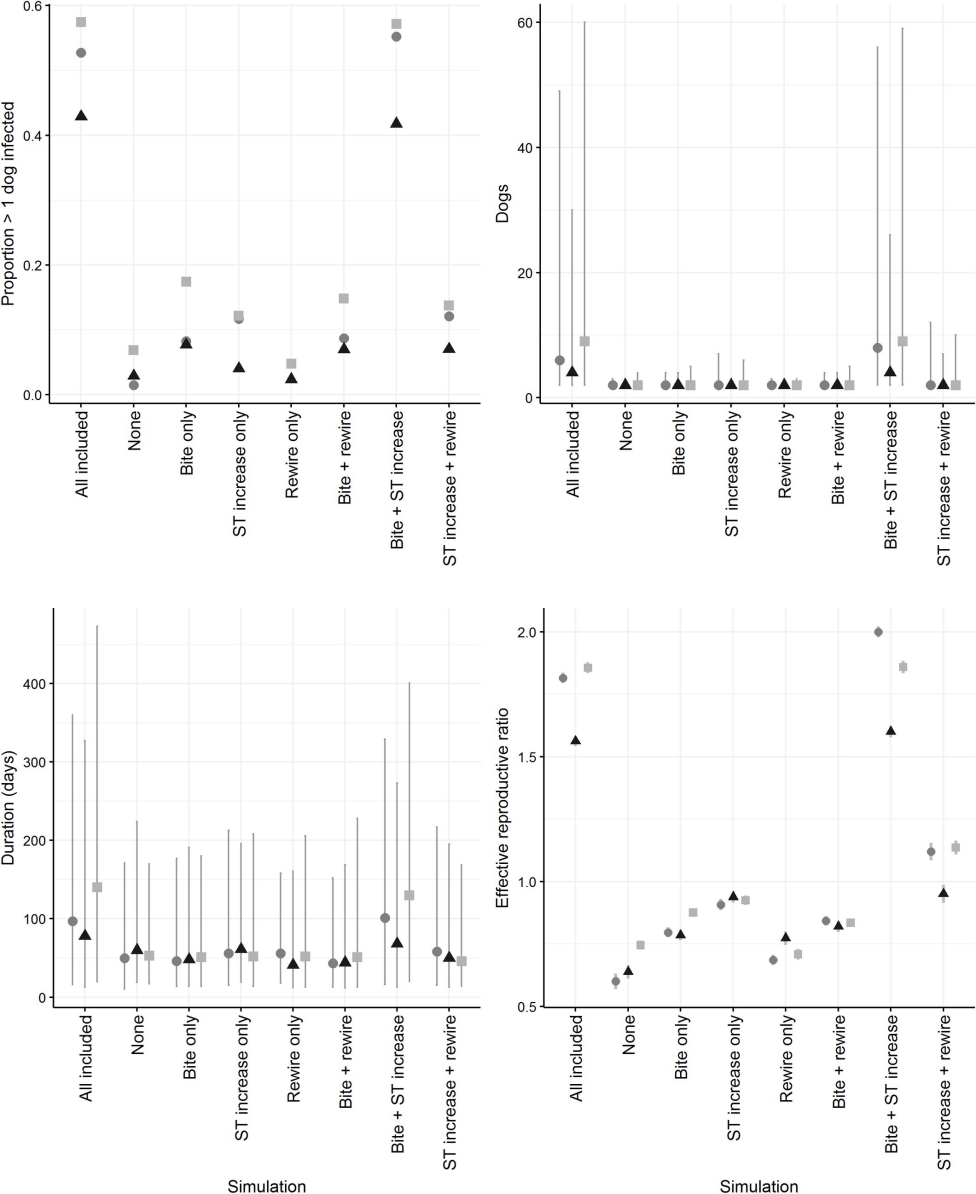
Proportion of predicted outbreaks in which > 1 dog was infected, and the number of infected dogs, outbreak duration and effective reproductive ratio in the first month following incursion, in a simulation model of rabies spread in networks of dog populations based on the empirical social networks of dogs in island communities in the Torres Strait, Australia. Simulations test the influence of inclusion of parameters associated with rabies-induced behavioural change (increased bite probability [associated with the furious form], increased spatio-temporal association [edge-weight; dumb form], wandering [‘re-wiring’; furious form]). Circle = Kubin, triangle = Saibai, square = Warraber. ST = spatio-temporal association. Grey lines = 95% range.

The proportion of outbreaks in which > 1 dog became infected, and the duration, number of infected dogs and *R*_*e*_ in the first month following incursion in simulations without all or with combinations of parameters for rabies-induced behavioural changes, are shown in Fig 3. Outputs from the simulation in each community with all parameters (increased bite probability [furious form], increased spatio-temporal association [edge-weight; dumb form], wandering [‘re-wiring’; furious form]) are included for comparison.

The simulation without parameters for rabies-induced behavioural changes (Fig 3; ‘None’) propagated following < 10% of incursions in all communities. In 95% of these predicted outbreaks, rabies spread to ≤ 3 other dogs during a median of ≤ 60 days. This was reflected in the low *R*_*e*_ estimate in the first month of these incursions (≤ 0.75).

Inclusion of one parameter associated with rabies-induced behavioural changes was still insufficient for sustained predicted outbreaks. Overall, < 20% incursions in these simulations resulted in rabies spread to ≤ 6 other dogs over a median duration of ≤ 56 days. *R*_*e*_ in the first month of these incursions indicated that increased spatio-temporal association, followed by an increased probability of bite were more likely to result in rabies spread than ‘re-wiring’ to increase network contacts in these simulations. This pattern was reflected in the upper 95% range of dogs infected, which was greatest when increased spatio-temporal association was included, and least when ‘re-wiring’ was included.

When combinations of rabies-induced behavioural changes were included, increased bite probability and spatio-temporal association together were sufficient to achieve similar proportions of predicted outbreaks in which > 1 dog was infected (40–60% of incursions) as the simulation with all parameters included (Fig 3 ‘Full’). Predicted impacts and *R*_*e*_ in the first month following incursion were also similar. *R*_*e*_ was greater than the sum of *R*_*e*_ from scenarios with increased bite probability and spatio-temporal association alone.

With combined spatio-temporal association and ‘re-wiring’, the 95% range of the number of infected dogs was greater than simulations in which only one parameter was included (up to 11 other dogs) but *R*_*e*_ in the first month following incursion was close to 1 in all communities, reflecting overall limited rabies spread. In the combined increased bite probability and ‘re-wiring’ simulation, propagation did not occur to > 4 dogs, reflecting the *R*_*e*_ of ≤ 0.8.

### The impact of pre-emptive vaccination

**Fig 4:**
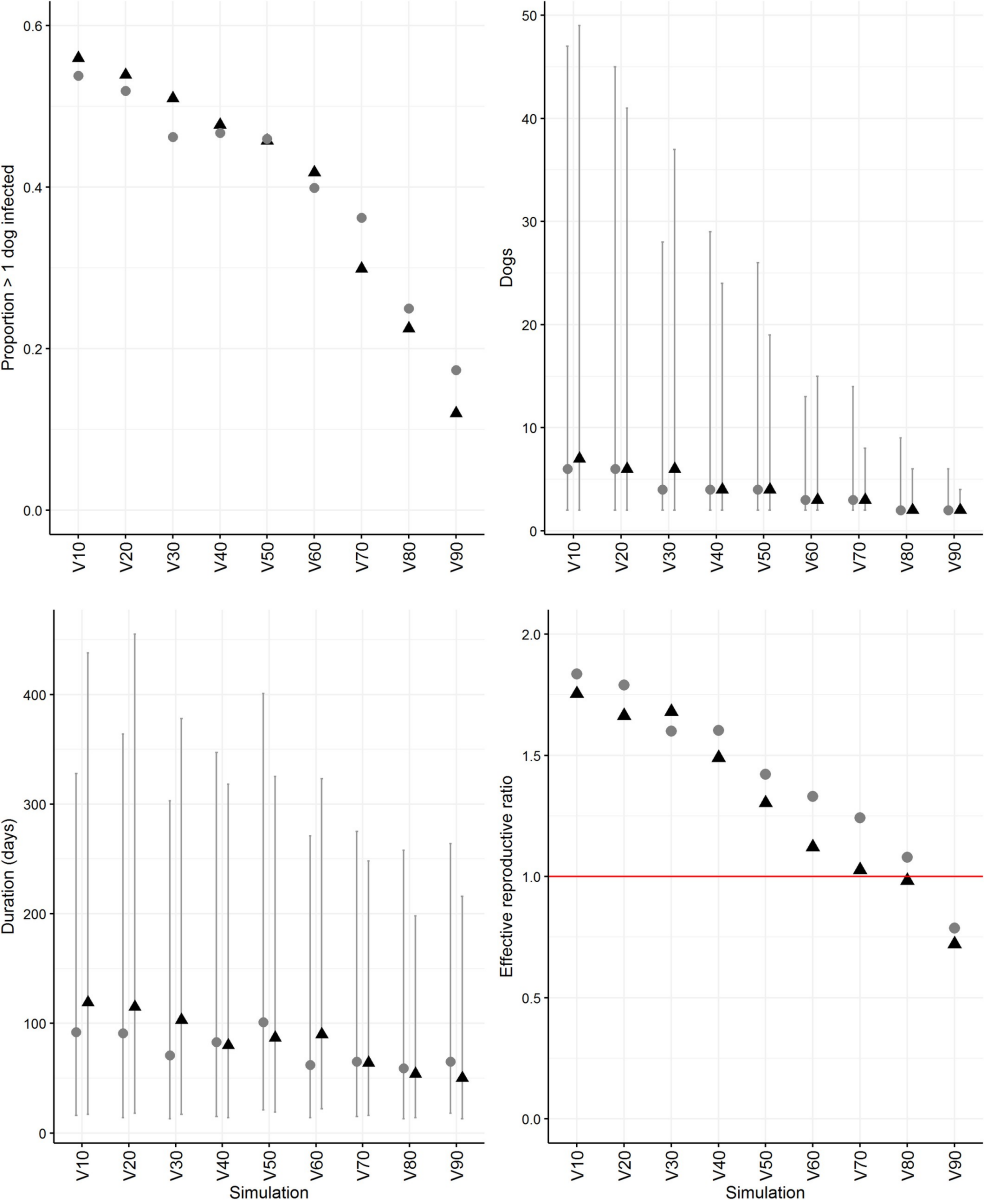
Proportion of predicted outbreaks in which > 1 dog was infected, and the number of infected dogs, outbreak duration and effective reproductive ratio in the first month following incursion in which >1 dog was infected, in a network simulation model of rabies spread based on the empirical social networks of dogs in Warraber, Torres Strait, Australia. Simulations test the influence of pre-emptive vaccination of increasing proportions of the population. Grey circles = simulations with births and deaths included, black triangles = simulations with births and deaths excluded. Grey lines = 95% range.

Due to the similarity between median outputs from each community and greatest variation in outputs from Warraber, only vaccination simulations using the Warraber network were included in this section. Initially, all parameters were included in these vaccination simulations (births and deaths were included). Vaccination simulations were then run without population turnover (births and deaths were excluded). Fig 4 shows all outputs.

In all simulations, the proportion of outbreaks in which > 1 dog was infected fell as the proportion of pre-emptively vaccinated dogs increased–a greater reduction was observed in the simulations without population turnover–and was < 40% when at least 70% of the population were vaccinated. The proportion of outbreaks in which more than one dog was infected was still 17% and 12% when 90% of the population were vaccinated in simulations with and without births and deaths, respectively.

In outbreaks in which > 1 dog was infected, the duration of outbreaks decreased as vaccination proportion increased (although the 95% range was always predicted > 195 days in all simulations). The median number of infected dogs was ≤ 3 once at least 60% of dogs were vaccinated in all simulations, but the 95% range was not consistently < 10 dogs until 80% and 70% of the population was vaccinated in simulations with and without births and deaths, respectively.

The median case rate was 1.6 cases/month (95% range 0.4–4.6 cases/month) when 70% of the population was vaccinated in simulations with births and deaths, with a median duration of 68 days (95% range 16–276 days). In simulations without births and deaths, the case rate was 1.4 cases/month (95% range 0.4–4.3 cases/month) when 70% of the population was vaccinated, with a median duration of 64 days (95% range 16–248 days). *R*_*e*_ estimated in the first month following incursion reflected these outputs. At ≥ 70% pre-emptive vaccination, *R*_*e*_ was approximately 1 or less when births and deaths were excluded. However, in the simulations with births and deaths *R*_*e*_ did not fall below 1 until > 80% of the population were pre-emptively vaccinated.

## Discussion

Our study is unique in that we modelled rabies spread in small populations of free-roaming dogs and incorporated the effect of rabies-induced behavioural changes. Key findings included the long duration of rabies persistence at low incidence in these populations, and the potential for outbreaks even with high levels of pre-emptive vaccination. This has implications for canine rabies surveillance, elimination and incursion prevention strategies, not only in rural areas with small communities, but also for elimination programs in urban areas. We discuss our findings and their implications below.

Without behavioural change, we could not achieve rabies propagation in the social networks in the current study; disruption of social contacts appears to be key for rabies maintenance in small populations of dogs. Social network studies have shown that dogs form contact-dense clusters [13, 20]. Increased bite probability and spatio-temporal association between contacts (edge-weight in the model) were most influential on rabies propagation in our model, but it is possible that ‘re-wiring’ of dogs is also influential in larger populations in which there is a greater probability that a dog would ‘re-wire’ to a completely new set of dogs in another cluster, thus increasing total contacts and enhancing spread (degree was also found to be highly influential on rabies spread). Ranges for these parameters were wide to reflect uncertainty which in turn reflects the difficulty of acquiring accurate field information about the behaviour of rabies-infected dogs. It is not ethical to allow dogs that have been identified in the clinical stages of rabies infection to continue to pose a threat to other animals and humans so that field data about contact behaviour can be collected. However, whilst these parameters were important for spread to occur, their wide range was not as influential on output variance relative to other parameters for which data were more certain. In the model, limiting types of behavioural change to each rabies form was a simplification that allowed us to differentiate the effects of types of network disruption. In reality, the association between rabies forms and behavioural changes is likely to be less distinct [33] and thus, rabies spread in small populations could be further enhanced if dogs display a range of behavioural changes.

Incubation period strongly influenced outbreak size and duration, and together with rabies-induced behavioural changes that enabled transmission, is likely to have resulted in the ‘slow-burn’ style of outbreaks (low incidence over long duration) that were predicted by this model. Within iterations in which propagation occurred, case rate was generally < 3 cases/month without vaccination, and 1.5 cases/month when 70% of dogs were pre-emptively vaccinated. At such low incidence, we believe that canine rabies is likely to have a low probability of detection in communities where there is high population turnover and aggressive free-roaming dogs can be normal [29, 43]. In these populations, dog deaths and fights between dogs are common. Undetected, slow-burn outbreaks in previously free regions are a great risk to humans because rabies awareness is likely to be low. They also provide more opportunity for latently infected dogs to travel between communities either by themselves, or with people, which could result in regional endemicity. Townsend et al (34) suggest a case detection rate of at least 5% (preferably 10%) is required to assess rabies freedom following control measures; surveillance capacity in rabies-free regions such as Oceania should be evaluated and enhanced if required.

Pre-emptive vaccination is another option to protect rabies-free regions; for example, an ‘immune-belt,’ an area in which dogs must be vaccinated, was established in the 1950s in northern Malaysia along the Thai border [44]. The World Health Organization recommends repeated mass parenteral vaccination of 70% of dog populations to achieve herd immunity [45]. Whilst the origin of this recommendation is unclear, it has been accepted for decades–for example, legislation allowed free-roaming of dogs in designated areas if at least 70% of the dog population was vaccinated in New York State in the 1940s [46]–and previous modelling studies of pre-emptive vaccination support this threshold [20, 47–49]. We found that vaccination with 70% coverage is expected result in outbreaks are self-limiting. Therefore, if inter-community dog movements are unlikely, the probability of regional spread is unlikely. However, given predicted upper 95% ranges of 8–14 rabies infected dogs for at least 8 months at 70% coverage, we recommend at least 90% coverage to reduce the effective monthly reproductive ratio < 1, limit human exposure, and provide a more certain barrier to regional spread, particularly in regions where dogs are socially and culturally connected to people and consequently, movement of dogs is likely.

In places in which movements are not easily restricted–such as urban centres in which dog populations are contiguous–our study indicates that comprehensive vaccination coverage is crucial and that reducing population turnover (for example, by increasing veterinary care to improve dog health) might not have a substantial effect on reducing the vaccination coverage required. The political and operational challenges of rabies elimination are well-documented [50], and lack of elimination or subsequent re-emergence is attributed to insufficient vaccination coverage (< 70% dog population overall, patchy coverage or insufficient duration [49, 51, 52]) and re-introduction of infected dogs [48, 53]. Pockets of unvaccinated dogs within well-vaccinated, urban areas could maintain rabies at a low incidence sufficient to re-introduce rabies as surrounding herd immunity wanes. It is also possible that with comprehensive, homogenous 70% coverage, a low incidence of rabies–such as appears possible at 70% vaccination in our study–is sufficient for endemicity in larger populations but is practically undetectable, giving the appearance of elimination. A higher proportion of vaccinated dogs might be required for elimination, and further modelling studies incorporating behavioural change in larger empirical networks are required to test this hypothesis.

Validation of a canine-rabies spread model is challenging, not only because variation between model outputs and observed data can arise from many sources, but because rabies surveillance is passive and case ascertainment is notoriously challenging [52], thus limiting the fitting of mathematical models and undermining comparison of predicted outputs to observed data. Mechanistic models are therefore a valuable tool to describe possible spread and develop hypotheses about rabies persistence, surveillance and control by using plausible, generalisable disease data (in the current study, the epidemiology of rabies) and context specific, ecological data (in the current study, empirical network data from small populations of dogs to provide contact rates). Although opportunity for validation is limited because outbreak data from small populations of dogs is scarce (and non-existent in our study area), observed patterns of disease spread (low incidence and long duration of outbreaks) are consistent with those predicted by the current study [37, 54]. Global sensitivity analysis indicated that population size (a parameter of reasonable certainty) and degree of connectivity had the greatest influence on duration, size and initial spread; this makes intuitive sense, and as expected, the largest and longest outbreaks were predicted in the Warraber network which had the highest median degree. Of the parameters that most influenced model outputs, parameterisation of the degree of connectivity was most likely to influence generalisability of our study findings because data are limited and social connectivity might vary between populations of free-roaming dogs. However, a study in N’Djaména, Chad, found that the average degree was 9 and 15 (maximum 20 and 64, respectively) in two populations of size 272 and 237 dogs, respectively [20], which is not dissimilar to the degree distribution of the small Torres Strait dog populations. Reassuringly, input parameters about which there was more uncertainty–for example, bite probabilities–were less influential on variation in outputs.

By exploring rabies epidemiology in small populations of free-roaming dogs–in which contact heterogeneity was determined in part by their social networks and in part by the disease–our study provides insights into how rabies-induced behavioural changes are important for endemicity of rabies in rural and remote areas. We found that rabies induced behavioural change is crucial for the disease to spread in these populations and enables a low incidence of rabies cases over a long duration. Without movement restrictions, we predict that substantially greater than the recommended 70% vaccination coverage is required to prevent rabies emergence in currently free areas.

## Supporting information

S1 Fig Convergence test plots using predicted duration of outbreak (days) and number of rabid dogs from a simulation model of rabies spread in free-roaming domestic dogs in Torres Strait communities, Queensland, Australia. a = coefficient of variation (CV) of sets of model simulations of increasing number of iterations. Horizontal red line = 0.025. b = proportion of values of < 0.025 (red line) for the coefficient of variation of the previous 100 sets of simulations for increasing numbers of iterations (x-axis). Horizontal line = 0.95.
(TIF)

S2 Fig First-order effect Sobol’ indices of parameters’ influence on the duration, number of rabies-infected dogs and mean monthly effective reproductive ratio of predicted rabies outbreaks following incursions of rabies in a simulated networks of dog populations based on the empirical networks of spatio-temporal association of dogs in three island communities in the Torres Strait, Australia. Cx = clinical signs, ST = spatio-temporal. Bars indicate 95% confidence intervals.
(TIF)
