# Supplementary figures and images for: Rabies-induced behavioural changes are key to rabies persistence in dog populations: Investigation using a network-based model
Source: PLoS Negl Trop Dis. 2019 Sep 23;13(9):e0007739. doi: 10.1371/journal.pntd.0007739 (PMC6776358; doi:10.1371/journal.pntd.0007739)

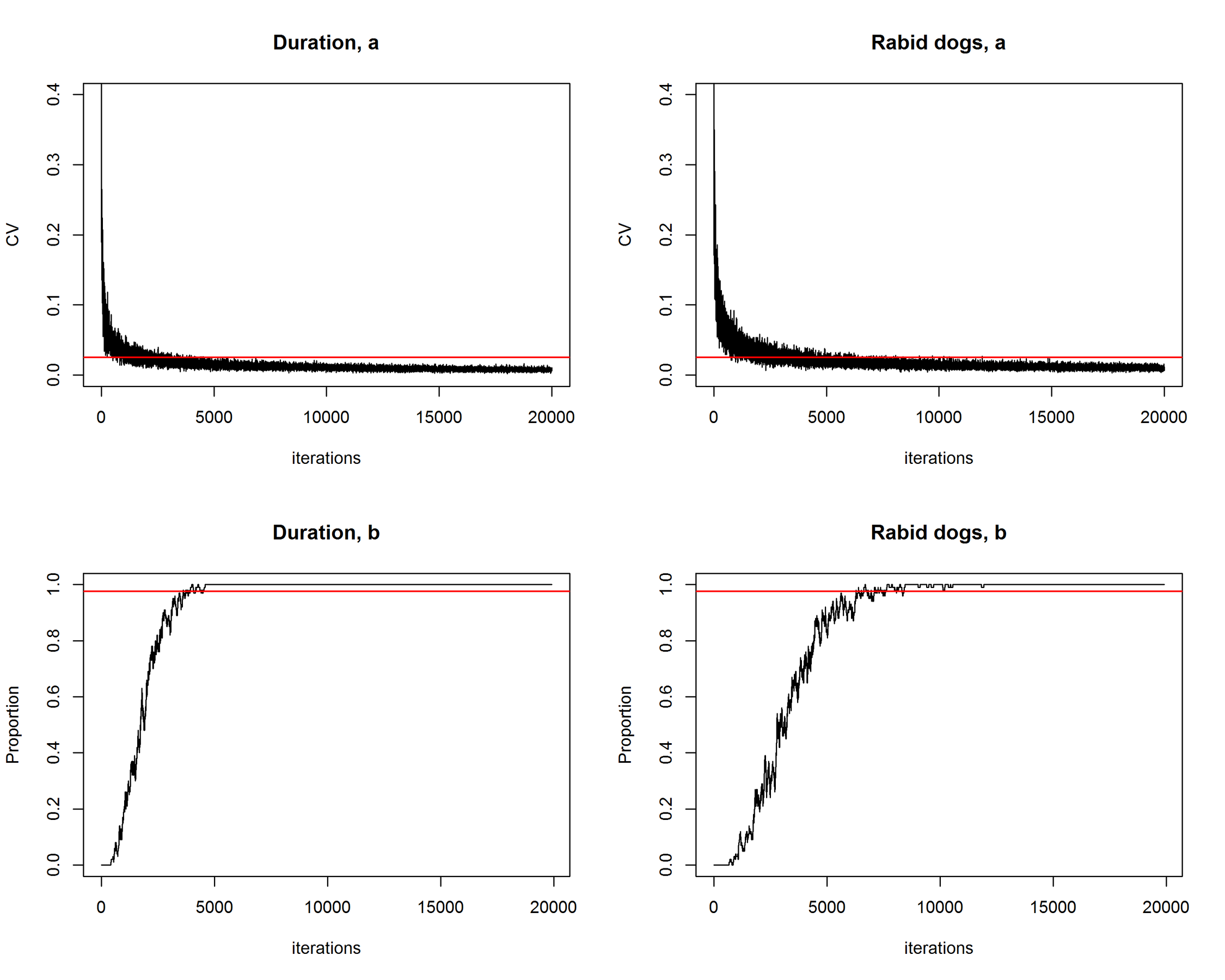

Supplement: S1 Fig — a = coefficient of variation (CV) of sets of model simulations of increasing number of iterations. Horizontal red line = 0.025. b = proportion of values of < 0.025 (red line) for the coefficient of variation of the previous 100 sets of simulations for increasing numbers of iterations (x-axis). Horizontal line = 0.95. (TIF) [file pntd.0007739.s001.tif]

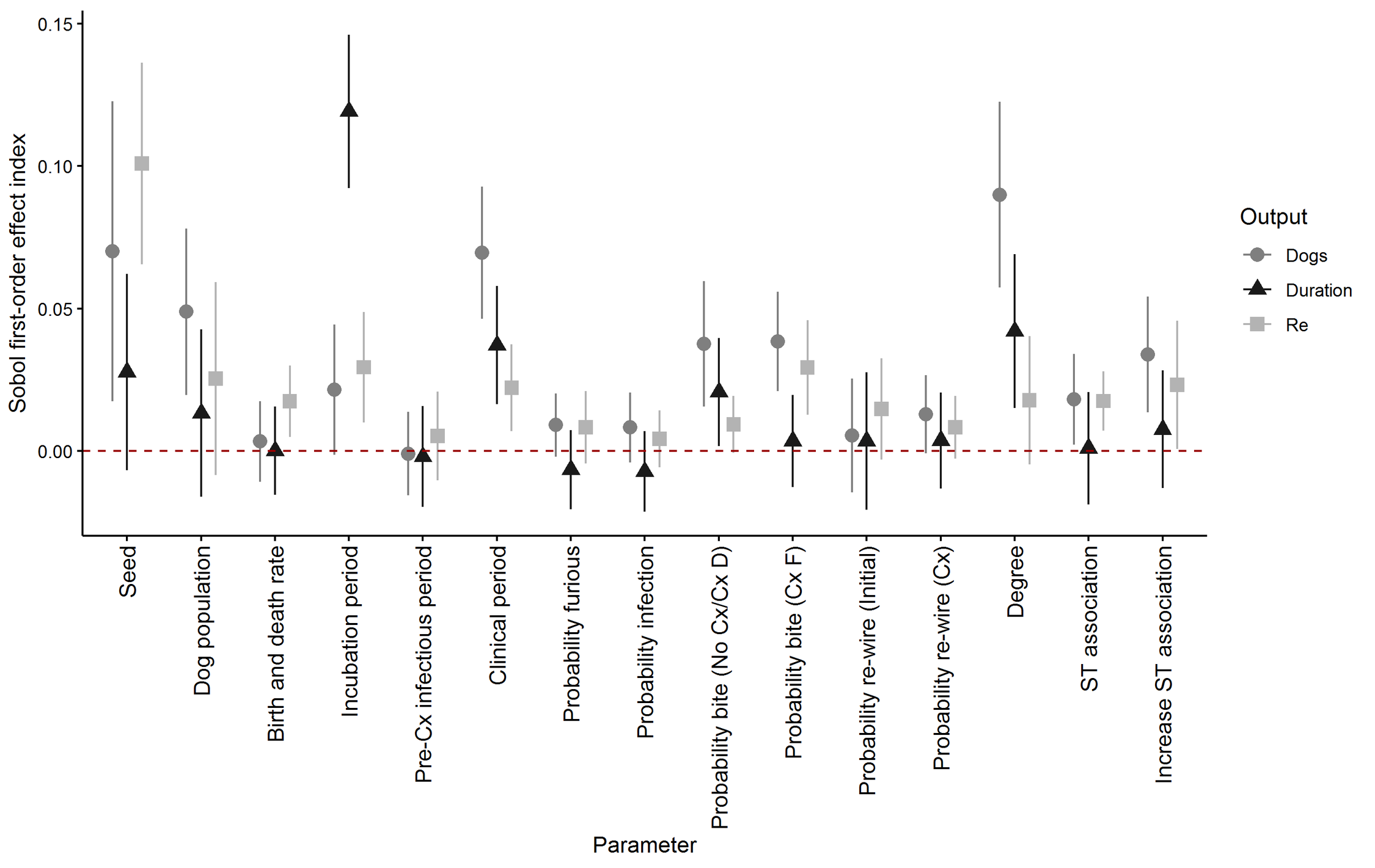

Supplement: S2 Fig — Cx = clinical signs, ST = spatio-temporal. Bars indicate 95% confidence intervals. (TIF) [file pntd.0007739.s002.tif]
